# Impact of pedigree completeness on genomic–polygenic evaluations for milk yield, fat percentage, and age at first calving in Thai multibreed dairy cattle

**DOI:** 10.5194/aab-69-455-2026

**Published:** 2026-08-27

**Authors:** Danai Jattawa, Praew Thiengpimol, Thanathip Suwanasopee, Mauricio A. Elzo, Thawee Laodim, Skorn Koonawootrittriron

**Affiliations:** 1 Department of Animal Science, Faculty of Agriculture, Kasetsart University, Bangkok 10900, Thailand; 2 Department of Agricultural Technology, Faculty of Science and Technology, Thammasat University, Pathum Thani 12120, Thailand; 3 Department of Animal Sciences, University of Florida, Gainesville, FL 32611-0910, USA; 4 Department of Animal Science, Faculty of Agriculture at Kamphaeng Saen, Kasetsart University Kamphaeng Saen Campus, Nakhon Pathom 73140, Thailand

## Abstract

Pedigree completeness is fundamental for accurate estimation of genetic parameters and reliable prediction of breeding values, yet incomplete pedigree records remain a persistent limitation in smallholder dairy systems, especially in multibreed populations. Although genomic data capture genetic relationships and compensate for missing pedigree information, the extent of this compensation under varying pedigree losses is not fully understood. This study evaluated the impact of pedigree completeness on genomic–polygenic evaluations of 305 d milk yield (MY), fat percentage (FP), and age at first calving (AFC) in the Thai multibreed dairy cattle population. Phenotypic records from 14 239 first-lactation cows and genotypes from 5175 animals imputed to 117 796 single-nucleotide polymorphisms (SNPs) were analyzed using a three-trait single-step genomic–polygenic model across nine scenarios (SO1–SO9). These ranged from full pedigree (SO1) to progressive exclusion of records for up to 50 % of phenotyped animals (SO9). Declining pedigree completeness altered variance structures, increasing additive genetic variance and heritability estimates for MY but reducing them for FP and AFC. Genomic–polygenic estimated breeding value (GPEBV) accuracies and ranking stability also declined with pedigree losses, particularly for FP and AFC, once more than 20 % of phenotyped animals lacked pedigree information. While genomic data partially compensated for moderate pedigree omissions, this capacity was trait-specific and dependent on the accuracy of imputation. The findings underscore that pedigree completeness remains indispensable for robust genomic–polygenic evaluations, particularly for low-heritability traits, where genomic information alone cannot ensure reliable selection. This study highlights the importance of systematic pedigree recording in smallholder-based tropical dairy systems to safeguard the accuracy of genetic evaluations and sustain long-term genetic improvement in multibreed populations.

## 1 Introduction

Accurate genetic evaluation is essential for improving economically important traits through enhanced selection precision and sustained genetic gain. Traditionally, polygenic models incorporating both pedigree and phenotypic information have been widely employed to estimate genetic parameters and predict breeding values (EBVs). The introduction of genomic technologies transformed genetic evaluation systems by enabling genomic–polygenic models that integrate genomic, pedigree, and phenotypic data. This integration has significantly increased the accuracy and reliability of selection decisions, leading to more effective genetic improvement strategies (VanRaden, 2008; Wiggans and Carrillo, 2020).

Despite these advances, the performance of genomic–polygenic models remains highly dependent on the quality and completeness of input data. In many smallholder or resource-constrained dairy production systems, the lack of infrastructure, technical support, and systematic record-keeping often results in incomplete pedigree data, thereby compromising the reliability of genetic evaluations (Thornton, 2010). In addition to missing records, pedigree recording errors may also occur under these conditions, leading to incorrect assignment of genetic relationships and further bias in parameter estimation. This limitation is especially pronounced in multibreed populations, where the accurate estimation of genetic relationships both within and across breeds and generations is critical to avoid biased parameter estimates, reduced genomic estimated breeding value (GEBV) accuracies, and unreliable rankings of selection candidates (Mulder and Bijma, 2005; Lourenco et al., 2015).

Genomic information provides an important complementary source of relationship information by capturing realized genetic relationships through dense single-nucleotide polymorphism (SNP) marker panels, enabling more accurate estimation of genetic relatedness by accounting for Mendelian sampling variation (Hayes et al., 2009; Misztal et al., 2009). However, the ability of genomic data to fully replace pedigree information remains context-dependent. Their effectiveness is influenced by several factors, including imputation accuracy, population structure, trait heritability, and the proportion of animals lacking pedigree records. Although several studies have reported the negative effects of missing or erroneous pedigree data on the estimation of genetic parameters and the accuracy of genomic predictions (Meyer, 2021; Pimentel et al., 2024), limited research has examined these effects in multibreed or smallholder systems, particularly in tropical dairy production environments.

The Thai dairy sector presents a relevant and timely case for investigating these challenges. This sector is characterized by a genetically diverse multibreed population (Koonawootrittriron et al., 2009) that is largely composed of smallholder farms (Rhone et al., 2008; Yeamkong et al., 2010), where maintaining complete pedigree records remains a persistent issue. While genomic technologies are increasingly available, their use in supporting reliable genetic evaluations under real-world pedigree limitations has yet to be evaluated in the Thai multibreed context.

Thus, this study assessed the impact of nine missing pedigree scenarios on genomic–polygenic evaluations for milk yield (MY), fat percentage (FP), and age at first calving (AFC) in the Thai multibreed dairy cattle population. Specifically, we investigated how these nine missing pedigree scenarios affected the estimation of genetic parameters, the accuracy of GEBVs, and the stability of animal rankings in the Thai dairy population. This research is expected to improve our understanding of the robustness of genomic–polygenic models in the Thai multibreed population and similar cattle populations composed of data-limited smallholder farms.

## 2 Materials and methods

### 2.1 Animals, data, and traits

Data were obtained from routine genomic–polygenic evaluations of the Thai multibreed dairy cattle population. The phenotypic file included records from 14 239 first-lactation cows, and the pedigree file comprised 25 551 animals, including 1749 sires and 23 802 dams. These cows calved between 1989 and 2019 and were located in 1312 farms spread across five regions of Thailand: Northern, Northeastern, Western, Central, and Southern.

The tropical climate of Thailand plays a crucial role in shaping dairy production systems, with two predominant monsoonal influences. The southwestern monsoon, occurring between May and September, brings substantial rainfall, whereas the northeastern monsoon, from October to February, results in cooler and drier conditions across most regions. These climatic variations create an environment characterized by elevated temperatures and high humidity that fluctuates seasonally and regionally. The average daily ambient temperature ranges from 26 to 29 °C, with relative humidity levels between 73 % and 76 %. During the summer season (March to June), temperatures range from 28.1 to 29.7 °C, with humidity levels between 63 % and 75 %. In the rainy season (July to October), temperatures fluctuate between 27.3 and 28.3 °C, with higher relative humidity levels ranging from 78 % to 81 %. In the winter season (November to February), temperatures vary between 23.4 and 26.7 °C and relative humidity between 69 % and 74 % (Thai Meteorological Department, 2025).

The multibreed structure of the Thai dairy cattle population resulted from long-term upgrading and systematic crossbreeding programs involving *Bos taurus* breeds (Holstein, Jersey, Brown Swiss, and Red Dane) and *Bos indicus* breeds (Brahman, Sahiwal, Red Sindhi, and Thai Native cattle). Both purebred and crossbred animals contributed to subsequent generations, producing an admixed population with genetic contributions from multiple ancestral breeds rather than discrete breed groups. This breeding strategy aimed to combine tropical adaptability derived primarily from indicine ancestry with enhanced milk production associated with taurine breeds (Koonawootrittriron et al., 2009). Consequently, the present population exhibits a continuous gradient of breed composition, with the majority of cows (92.75 %) carrying Holstein fractions of 75 % or greater.

The traits evaluated included 305 d milk yield (MY, kg), 305 d fat percentage (FP, %), and age at first calving (AFC, months). Milk production records were collected monthly starting from the fifth day after calving until completion of lactation. Only cows with the first test-day record before 40 d in milk and with at least four test-day records (94 to 141 d in milk) were retained for analysis. The last test day considered was the eleventh record, collected between 299 and 352 d in milk. The MY was estimated using the test interval method based on test-day milk yield records (Sargent et al., 1968; Koonawootrittriron et al., 2001). The FP was calculated as the average of test-day fat percentage values recorded from calving up to 305 d in milk. The AFC was defined as the interval, in months, between birth and the date of first calving.

### 2.2 DNA samples and genotypes

Blood and semen samples were collected from 5003 animals (184 sires and 4819 cows) across 475 farms. DNA was extracted using the MasterPure™ DNA purification kit (Epicentre ^®^, Madison, WI, USA), and DNA quality and concentration were assessed using a NanoDrop™ 2000 spectrophotometer (Thermo Scientific, Wilmington, DE, USA). Samples were considered suitable for genotyping if their DNA concentration exceeded 15 ng µ L^−1^ and had an absorbance ratio of approximately 1.8 at 260/280 nm. The selected DNA samples were subsequently sent to GeneSeek Inc. (Lincoln, NE, USA) for genotyping.

The genotyped animals originated from routine genomic–polygenic evaluation programs implemented in the Thai dairy cattle population. The allocation of genotyping chips was determined by three key factors: (1) pedigree relationships, where closely related individuals were prioritized for high-density (HD) genotyping, while lower-density (LD) chips were used for other animals; (2) availability of genotyping chips at different time points (for instance, GGP80K was used as the HD chip in 2013, whereas GGP150K and GGP100K were considered as HD and LD chips, respectively, from 2021 onward); and (3) budgetary constraints, which determined the number of animals genotyped annually. As a result, animals were genotyped using various GGP chips, including GGP9K ( n=1412 ), GGP20K ( n=570 ), GGP26K ( n=540 ), GGP30K ( n=563 ), GGP50K ( n=887 ), GGP80K ( n=139 ), GGP100K ( n=529 ), and GGP150K ( n=363 ). The number of SNP markers on each chip was 8810 for GGP9K, 19 720 for GGP20K, 26 151 for GGP26K, 30 106 for GGP30K, 47 843 for GGP50K, 76 883 for GGP80K, 95 256 for GGP100K, and 139 376 for GGP150K.

To ensure consistency in marker density, animals genotyped with GGP9K, GGP20K, GGP26K, GGP30K, GGP50K, GGP80K, and GGP100K were imputed to GGP150K using the combined family- and population-based imputation approach implemented in Findhap 4 (VanRaden and Sun, 2014). This algorithm reconstructs haplotypes by integrating pedigree transmission information with population linkage disequilibrium patterns. In addition to harmonizing marker density across genotyping platforms, the method allows genotype inference for closely related non-genotyped animals when sufficient pedigree connectivity is available (VanRaden et al., 2015). In the present population, a subset of non-genotyped parent animals showed strong genetic connections with genotyped relatives, particularly through multiple genotyped progenies. These relationships provided sufficient inheritance information to infer parental haplotypes from chromosomal segments shared among offspring. Consequently, genomic information was obtained for 172 previously non-genotyped animals (163 sires and 9 dams; previous work in this population demonstrated that incorporating imputed genotypes from closely related animals improves the accuracy of genomic–polygenic evaluation; Jattawa et al., 2025). Consequently, these additional 172 animals were included in the study.

Imputation accuracy was not re-estimated in the current dataset because masked validation genotypes were not available for all animals. However, the same Findhap pipeline has previously been validated in the Thai multibreed dairy population, showing average allelic concordance exceeding 0.84 across GGP SNP platforms and producing genomic evaluation results comparable to those obtained using true genotypes (Jattawa et al., 2016). Following imputation, SNP markers with a minor allele frequency below 0.05 or a call rate lower than 0.90 were excluded. Thus, the final genotypic dataset comprised 5175 animals (341 sires and 4834 cows), with all genotyped cows having corresponding phenotypic records, and a total of 117 796 actual and imputed SNP markers.

### 2.3 Scenarios and genomic–polygenic evaluations

Pedigree information from the Thai dairy cattle population was used to construct nine scenarios (SO) for implementing genomic–polygenic evaluations. The scenarios were as follows: SO1 contained all available pedigree information; SO2 removed sire identification for all genotyped animals; SO3 removed dam identification for all genotyped animals; SO4 removed both sire and dam identification for all genotyped animals; and SO5 to SO9 excluded pedigree information for 10 %, 20 %, 30 %, 40 %, and 50 % of animals with phenotypic records, respectively. Numbers of animals in the phenotypic and pedigree files as well as numbers of sires and dams in each scenario (SO1 to SO9) are shown in Table 1.

**Table 1:** Numbers of animals across nine missing pedigree scenarios.

Numbers of animals	Missing pedigree scenario^*^								
	SO1	SO2	SO3	SO4	SO5	SO6	SO7	SO8	SO9
Number of animals with phenotypes	14 239	14 239	14 239	14 239	14 239	14 239	14 239	14 239	14 239
Number of animals with genotypes	5175	5175	5175	5175	5175	5175	5175	5175	5175
Number of animals in pedigree	25 551	25 304	22 687	22 440	23 759	22 015	21 858	20 585	19 179
Number of sires	1749	1502	1749	1502	1585	1664	1410	1499	1324
Number of cows	23 802	23 802	20 938	20 938	22 174	20 351	20 448	19 086	17 855

^*^ SO1: all available pedigree information; SO2: sire identification removed for all genotyped animals; SO3: dam identification removed for all genotyped animals; SO4: both sire and dam identification removed for all genotyped animals; SO5: no pedigree information for 10 % of animals with phenotypes; SO6: no pedigree information for 20 % of animals with phenotypes; SO7: no pedigree information for 30 % of animals with phenotypes; SO8: no pedigree information for 40 % of animals with phenotypes; SO9: no pedigree information for 50 % of animals with phenotypes.

These nine scenarios were designed to reflect practical challenges commonly encountered in genomic–polygenic evaluations, where pedigree information is often incomplete, inaccurate, or missing for specific subsets of the population. SO1 represented the optimal case in which all available pedigree and genomic data were used. Nevertheless, pedigree information in SO1 remained partially incomplete in this Thai smallholder dataset, with 609 phenotyped animals (4.28 %) lacking dam identification, whereas all genotyped animals had complete parental identification. SO2 to SO4 progressively removed pedigree information from specific genotyped groups to assess which categories of animals should be prioritized for genotyping when pedigree data and financial resources are limited. These scenarios aimed to simulate decision-making conditions in breeding programs where selecting animals for genotyping must be strategic to maximize the benefit of genomic evaluations. In contrast, SO5 to SO9 simulated varying levels of random pedigree data loss among animals with phenotypic records. For SO5–SO9, pedigree removal followed a controlled nested (cumulative) design rather than independent random sampling. First, a random subset of animals was selected in SO5, and their pedigree information was removed. The same animals remained without pedigree in subsequent scenarios, while additional randomly selected animals were added progressively in SO6, SO7, SO8, and SO9. Consequently, each subsequent scenario contained all animals from the previous scenario plus newly added animals with removed pedigree information. This approach ensured that increasing levels of missing pedigree represented incremental expansions of the same affected population, thereby maintaining comparability among scenarios and avoiding variation caused by repeated random re-sampling of different animal groups or breed compositions. These scenarios were constructed to evaluate the extent to which animals with incomplete pedigree information could still be incorporated into genomic–polygenic evaluations without substantially compromising the accuracy of parameter estimation and breeding value prediction. This reflects real-world conditions in large-scale evaluations, where a proportion of historical pedigree records may be missing or unreliable.

Phenotypic, pedigree, and genotypic data were used to obtain variance–covariance components, genetic parameters, and genomic–polygenic estimated breeding values (GPEBVs) for MY, FP, and AFC in the nine missing pedigree scenarios. Variance–covariance estimates were computed using a three-trait single-step genomic–polygenic model (Aguilar et al., 2010). The model included contemporary group (herd–year–season), calving age (including only for MY and FP), and heterosis as fixed effects and additive genetic and residual as random effects. Contemporary group was fitted as a categorical fixed effect (6626 levels). Calving age was fitted as a linear covariate (15 to 60 months) for MY and FP to account for variation in physiological maturity at first calving. Age at first calving (AFC) was analyzed as a response trait and was therefore not included as a fixed effect in the AFC model. Heterosis was fitted as a continuous covariate (0 % to 100 %) to capture non-additive genetic effects arising from crossbreeding.

An additive breed effect (e.g., Holstein proportion or breed group) was not fitted because the Thai dairy population represents a continuous multibreed admixture formed through long-term upgrading toward Holstein rather than clearly separable breed classes. Under this structure, additive differences associated with breed composition are accounted for through the pedigree and genomic relationship matrices, with breeding values expressed relative to the population mean. In contrast, heterosis was explicitly modeled to capture non-additive genetic effects arising from crossbreeding. Heterosis was considered to be a linear function of breed heterozygosity between Holstein and other breeds, calculated as the sum of products of breed fractions from the sire and the dam: (1) the Holstein fraction of the sire was multiplied by the other-breed fraction of the dam, and (2) the other-breed fraction of the sire was multiplied by the Holstein fraction of the dam. The random effects in the model were additive genetic and residual, each assumed to have zero mean and an equal variance–covariance matrix for all animals in the population regardless of their breed composition.

The variance–covariance matrix of additive genetic effects was defined as $H⊗V_{a}$ , where **H** represented the genomic–polygenic additive relationship matrix; $V_{a}$ was the 3×3 matrix of additive genomic–polygenic variances and covariances for MY, FP, and AFC; and ⊗ denoted the Kronecker product. The **H** matrix was formulated as

1 $$\left[\begin{array}{cc} A_{11}+A_{12}A_{22}^{-1}(G_{22}-A_{22})A_{22}^{-1}G_{21} & A_{12}A_{22}^{-1}G_{22}\\ G_{22}A_{22}^{-1}A_{21} & G_{22} \end{array}\right],$$

where $A_{11}$ was the submatrix of additive genetic relationships among non-genotyped animals, $A_{12}$ represented additive genetic relationships between non-genotyped and genotyped animals, $A_{22}^{-1}$ was the inverse of the additive genetic relationship submatrix for genotyped animals, and $G_{22}$ was the genomic relationship matrix among genotyped individuals (Legarra et al., 2009). The $G_{22}$ matrix was computed as $\frac{{\mathrm{ZZ}}^{\prime }}{2\sum p_{j}(1-p_{j})}$ , where $p_{j}$ represented the frequency of allele 2 in locus j , and the elements of matrix **Z** were computed as $z_{ij}=(0-2p_{j}$ ) for genotype = 11 in locus j , $z_{ij}=(1-2p_{j})$ for genotype = 12 and 21 in locus j , and $z_{ij}=(2-2p_{j})$ for genotype = 22 in locus j (VanRaden, 2008; Aguilar et al., 2010). To ensure compatibility between pedigree and genomic information in the single-step framework, the $G_{22}$ matrix was scaled to be compatible with $A_{22}$ , using the default settings in the BLUPF90 Family of Programs (Misztal et al., 2002); i.e., the means of the diagonal and off-diagonal elements of $G_{22}$ were equal to those of $A_{22}$ . The residual variance–covariance matrix was equal to $I⊗V_{e}$ , where **I** denoted the identity matrix, and $V_{e}$ was the 3×3 matrix of residual variances and covariances among traits.

The average information restricted maximum likelihood (AIREML) method, implemented in the AIREMLF90 software (Tsuruta, 2014), was used to estimate variance and covariance components for MY, FP, and AFC. Because variance components were estimated using the AIREML algorithm, moderate variation among scenarios is expected when relationship structures change, particularly under reduced pedigree connectivity where the balance between pedigree- and genome-derived information in the **H** matrix is altered. Standard errors for additive genetic and environmental variances were derived from the inverse of the average information matrix. Phenotypic variances, covariances, heritabilities, and their standard errors were computed with the repeated sampling procedure available in the AIREMLF90 program (Meyer and Houle, 2013). The repeated sampling procedure was also used to obtain phenotypic, additive genetic, and environmental correlations between MY, FP, and AFC, along with their corresponding standard errors. These represent model-based (observed) standard errors conditional on each pedigree scenario and were used to compare parameter stability across scenarios.

Genomic–polygenic estimated breeding values (GPEBVs) for MY, FP, and AFC were computed using the single-step genomic BLUP (ssGBLUP) procedure contained in the BLUPF90 software (Misztal et al., 2002; Aguilar et al., 2010). Estimates of variance–covariance components and the **H** matrix from the nine missing pedigree scenarios were incorporated into the ssGBLUP analyses. Pearson correlations were calculated to evaluate agreement among GPEBVs, whereas Spearman rank correlations were used to assess the consistency of animal rankings between the reference scenario (SO1) and each alternative scenario (SO2 to SO9). Correlations were calculated using the CORR procedure in SAS^®^ OnDemand for Academics (SAS Institute Inc., Cary, NC, USA).

## 3 Results and discussion

### 3.1 Effect of incorporating incomplete pedigree data on variance–covariance components

The additive genetic variances and covariances for MY, FP, and AFC estimated using the three-trait single-step genomic–polygenic model across the nine missing pedigree scenarios are presented in Table 2. The pattern of pedigree exclusion influenced variance component estimates differently among traits. As pedigree completeness decreased, additive genetic variance for MY showed an overall tendency toward larger estimates, although fluctuations occurred among intermediate scenarios, increasing from 143550±22493 kg^2^ (SO6) to 177440±16357 kg^2^ (SO9). These non-monotonic changes reflect adjustments in the relative contribution of pedigree and genomic relationships within the single-step framework rather than a linear response to pedigree loss. Additive genetic variance for FP remained relatively stable across scenarios, indicating limited sensitivity to pedigree perturbation. In contrast, additive genetic variance for AFC varied across scenarios and was generally lower under several incomplete pedigree conditions compared with SO1 ( 3.75±0.73 month^2^), with the lowest estimate observed in SO7 ( 1.72±0.74 month^2^).

**Table 2:** Additive genetic variances and covariances for 305 d milk yield (MY), 305 d fat percentage (FP), and age at first calving (AFC) estimated using a three-trait single-step genomic–polygenic model across nine missing pedigree scenarios.

Variance component		Missing pedigree scenario^*^								
		SO1	SO2	SO3	SO4	SO5	SO6	SO7	SO8	SO9
Var (MY),	Var	147 570.00	151 120.00	152 450.00	150 970.00	155 300.00	143 550.00	149 450.00	161 640.00	177 440.00
kg^2^	SE	21 102.00	22 720.00	22 120.00	22 894.00	23 563.00	22 493.00	25 485.00	25 746.00	16 357.00
Cov (MY, FP),	Cov	- 33.01	- 37.02	- 32.48	- 38.39	- 42.47	- 36.27	- 47.50	- 44.41	- 47.54
kg %	SE	12.02	12.76	12.69	12.92	13.36	13.11	14.47	14.91	4.69
Cov (MY, AFC),	Cov	- 62.81	- 130.04	- 110.92	- 150.45	- 91.15	- 29.67	- 31.49	83.64	54.14
kg × month	SE	87.95	91.71	90.28	91.36	92.19	91.41	99.45	103.23	41.36
Var (FP),	Var	0.05	0.04	0.05	0.04	0.04	0.05	0.03	0.04	0.02
%^2^	SE	0.01	0.01	0.01	0.01	1.39	0.01	0.02	0.02	0.01
Cov (FP, AFC),	Cov	0.11	0.12	0.11	0.12	0.08	0.10	0.05	0.03	- 0.08
% × month	SE	0.07	0.07	0.07	0.07	0.07	0.07	0.08	0.08	0.01
Var (AFC),	Var	3.75	3.17	3.35	2.92	2.18	2.32	1.72	2.29	2.03
month^2^	SE	0.73	0.73	0.73	0.72	0.70	0.73	0.74	0.80	0.23

^*^ SO1: all available pedigree information; SO2: sire identification removed for all genotyped animals; SO3: dam identification removed for all genotyped animals; SO4: both sire and dam identification removed for all genotyped animals; SO5: no pedigree information for 10 % of animals with phenotypes; SO6: no pedigree information for 20 % of animals with phenotypes; SO7: no pedigree information for 30 % of animals with phenotypes; SO8: no pedigree information for 40 % of animals with phenotypes; SO9: no pedigree information for 50 % of animals with phenotypes.

The results indicated that the completeness of pedigree information influenced the estimates of additive genetic variances, with differing responses across MY, FP, and AFC. The overall increase in additive genetic variance estimates for MY under incomplete pedigree scenarios likely reflects a redistribution of genetic variance as pedigree relationships were reduced, altering the relative contribution of pedigree- and genome-derived information within the single-step model. Similar behavior has been reported when pedigree links are removed, leading to changes in variance partitioning and genetic evaluation outcomes (Elzo et al., 2015). Greater reliance on genomic relationships when pedigree information was missing may have contributed to this pattern, as genomic relationships alone may not fully capture all additive genetic relationships present in the population (Meyer, 2021).

In contrast, the estimates of additive genetic variance for FP remained relatively stable across all pedigree scenarios, suggesting that this trait was less sensitive to missing pedigree information. The stability of the FP additive genetic variance in this study may be attributed to the FP genetic architecture, which may have allowed genomic information to more effectively compensate for the absence of pedigree relationships. Conversely, the estimates of the additive genetic variance for AFC showed greater variability across pedigree scenarios and were generally reduced under several incomplete pedigree conditions, indicating higher sensitivity to missing pedigree information. This pattern agrees with the nature of fertility traits, which generally have lower heritability (Liu et al., 2008; Berry et al., 2013; Shao et al., 2021; Kgari et al., 2022) and are, therefore, more susceptible to pedigree errors and incompleteness of pedigree information. Pimentel et al. (2024) demonstrated that pedigree inaccuracies can introduce biases and reduce the accuracy of estimated breeding values, with particularly pronounced effects in low-heritability traits. These findings are consistent with the present results, which suggest that genomic information alone may not sufficiently compensate for missing pedigree data in traits like AFC.

Table 3 presents the environmental variances and covariances for MY, FP, and AFC estimated across the nine missing pedigree scenarios. Environmental variance estimates varied across scenarios without following a strictly monotonic pattern, indicating that the observed changes were associated with redistribution of variance components under altered pedigree connectivity rather than a linear response to pedigree loss. For MY, estimated environmental variances ranged from 438350±18995 kg^2^ in SO1 to 405970±16581 kg^2^ in SO9, with intermediate scenarios showing moderate fluctuations. Estimates for FP remained relatively stable, varying within a narrow interval from 0.20±0.01 %^2^ to 0.23±0.00 %^2^. In contrast, AFC showed greater variability, ranging from 17.32±0.68 month^2^ (SO1) to 18.79±0.39 month^2^ (SO9).

**Table 3:** Environmental variances and covariances for 305 d milk yield (MY), 305 d fat percentage (FP), and age at first calving (AFC) estimated using a three-trait single-step genomic–polygenic model across nine missing pedigree scenarios.

Variance component		Missing pedigree scenario^*^								
		SO1	SO2	SO3	SO4	SO5	SO6	SO7	SO8	SO9
Var (MY),	Var	438 350.00	435 750.00	434 520.00	435 500.00	430 680.00	439 760.00	433 840.00	421 410.00	405 970.00
kg^2^	SE	18 995.00	20 350.00	19 812.00	20 595.00	21 262.00	20 731.00	23 541.00	23 842.00	16 581.00
Cov (MY, FP),	Cov	- 26.64	- 22.95	- 27.26	- 21.50	- 17.56	- 22.89	- 12.12	- 14.94	- 11.63
kg %	SE	11.47	12.17	11.96	12.32	12.76	12.65	14.12	14.49	7.25
Cov (MY, AFC),	Cov	227.10	286.58	270.46	305.54	253.09	198.63	199.18	92.08	119.54
kg × month	SE	80.82	84.62	83.15	84.97	86.88	87.23	95.56	98.77	42.52
Var (FP),	Var	0.20	0.20	0.20	0.20	0.21	0.20	0.22	0.21	0.23
%^2^	SE	0.01	0.01	0.01	0.01	1.35	0.01	0.02	0.02	0.00
Cov (FP, AFC),	Cov	- 0.10	- 0.11	- 0.10	- 0.12	- 0.08	- 0.10	- 0.05	- 0.03	0.07
% × month	SE	0.07	0.07	0.07	0.07	0.07	0.08	0.08	0.08	0.04
Var (AFC),	Var	17.32	17.84	17.67	18.05	18.71	18.57	19.10	18.57	18.79
month^2^	SE	0.68	0.70	0.69	0.69	0.69	0.72	0.75	0.80	0.39

^*^ SO1: all available pedigree information; SO2: sire identification removed for all genotyped animals; SO3: dam identification removed for all genotyped animals; SO4: both sire and dam identification removed for all genotyped animals; SO5: no pedigree information for 10 % of animals with phenotypes; SO6: no pedigree information for 20 % of animals with phenotypes; SO7: no pedigree information for 30 % of animals with phenotypes; SO8: no pedigree information for 40 % of animals with phenotypes; SO9: no pedigree information for 50 % of animals with phenotypes.

In single-step genomic–polygenic evaluation, environmental and additive genetic variances are estimated jointly, and reductions in pedigree information modify the balance between pedigree- and genome-derived relationships within the combined relationship matrix (**H**; Legarra et al., 2009). Consequently, moderate scenario-to-scenario fluctuations are expected as variance components are redistributed between additive genetic and residual effects. This redistribution primarily arises from the loss of pedigree-based covariances among animals when pedigree links are removed, with stronger effects observed for non-genotyped individuals, whereas relationships among genotyped animals remain largely preserved through genomic information. The relatively lower MY environmental variance estimates and higher AFC environmental variance estimates observed under several incomplete pedigree scenarios are therefore consistent with compensatory reallocation of variance components rather than reflecting systematic biological trends. Comparable sensitivity of variance component estimates has been reported when incomplete pedigree information alters relationship structure and variance partitioning in genomic evaluations (Masuda et al., 2021), reflecting known properties of genomic–pedigree integration when pedigree connectivity is reduced.

Despite these changes in variance partitioning, phenotypic variances remained highly stable across MY, FP, and AFC (Table 4). Because identical phenotypic records were used in all scenarios, only minor differences were observed, arising from re-estimation of variance components under altered pedigree connectivity. The phenotypic variance for MY ranged from 583050±9988 kg^2^ (SO8) to 586960±10256 kg^2^ (SO3), whereas only small changes occurred for FP (from 0.24±0.00 %^2^ (SO9) to 0.25±0.01 %^2^ (SO1)) and AFC (from 20.82±0.34 month^2^ (SO9) to 21.07±0.36 month^2^ (SO1)), indicating only marginal differences in overall trait variability. This stability indicates that incomplete pedigree information primarily affected the partitioning between additive genetic and residual components rather than the total observed variability in the traits.

**Table 4:** Phenotypic variances and covariances for 305 d milk yield (MY), 305 d fat percentage (FP), and age at first calving (AFC) estimated using a three-trait single-step genomic–polygenic model across nine missing pedigree scenarios.

Variance component^b^		Missing pedigree scenario^a^								
		SO1	SO2	SO3	SO4	SO5	SO6	SO7	SO8	SO9
Var (MY),	Var	585 920.00	586 870.00	586 960.00	586 470.00	585 990.00	583 310.00	583 290.00	583 050.00	583 410.00
kg^2^	SE	10 203.00	10 268.00	10 256.00	10 233.00	10 202.00	10 030.00	10 029.00	9 988.00	9 530.80
Cov (MY, FP),	Cov	- 59.66	- 59.96	- 59.74	- 59.90	- 60.03	- 59.15	- 59.61	- 59.35	- 59.17
kg %	SE	6.50	6.51	6.54	6.50	6.49	6.44	6.14	6.40	6.16
Cov (MY, AFC),	Cov	164.30	156.54	159.54	155.10	161.94	168.97	167.69	175.72	173.68
kg × month	SE	43.22	43.16	43.19	42.94	42.56	42.21	41.97	41.97	36.08
Var (FP),	Var	0.25	0.25	0.25	0.25	0.25	0.25	0.25	0.25	0.24
%^2^	SE	0.01	0.01	0.01	0.01	0.61	0.01	0.01	0.04	0.00
Cov (FP, AFC),	Cov	0.01	0.01	0.01	0.01	0.01	0.01	0.00	0.00	- 0.01
% × month	SE	0.04	0.04	0.04	0.04	0.04	0.04	0.04	0.04	0.04
Var (AFC),	Var	21.07	21.01	21.02	20.97	20.89	20.89	20.83	20.85	20.82
month^2^	SE	0.36	0.36	0.36	0.36	0.35	0.35	0.35	0.35	0.34

^a^ SO1: all available pedigree information; SO2: sire identification removed for all genotyped animals; SO3: dam identification removed for all genotyped animals; SO4: both sire and dam identification removed for all genotyped animals; SO5: no pedigree information for 10 % of animals with phenotypes; SO6: no pedigree information for 20 % of animals with phenotypes; SO7: no pedigree information for 30 % of animals with phenotypes; SO8: no pedigree information for 40 % of animals with phenotypes; SO9: no pedigree information for 50 % of animals with phenotypes. ^b^ Repeated sampling approach of Meyer and Houle (2013).

The impact of missing pedigree data on variance and covariance estimates varied depending on whether the exclusions affected genotyped or non-genotyped animals. When pedigree information was absent only for genotyped animals (SO2 to SO4), variance estimates remained stable for all traits, with deviations from SO1 (0.00 % to 22.13 %) being small relative to their associated standard errors, indicating minimal practical differences among these scenarios. This suggested that genomic information partially compensated for the absence of pedigree relationships, agreeing with the observations of Masuda et al. (2021), who demonstrated that the incorporation of unknown parent groups (UPGs) in ssGBLUP models preserved genetic relationship structures despite incomplete pedigree information. However, as pedigree incompleteness extended to non-genotyped animals (SO5 to SO9), variance estimates exhibited greater instability, with the most pronounced deviations occurring in SO9 ( - 60.00 % to 23.61 %). This pattern indicated that the model became increasingly reliant on genomic information, which alone was insufficient to fully capture additive genetic relationships, thereby leading to a greater degree of variance fluctuation.

Additionally, the impact of missing pedigree information was more pronounced when both parental records were absent (SO4) than when only sire (SO2) or dam (SO3) information was unavailable for genotyped animals. The deviations observed in SO4 ranged from - 41.87 % to 8.19 %, exceeding those in SO2 ( - 21.87 % to 2.91 %) and SO3 ( - 31.20 % to 1.99 %). These results demonstrate that the absence of both parental records had the greatest influence on variance inflation, with the lack of dam information contributing slightly more to deviations than the absence of sire data. This underscores the importance of prioritizing animals with at least one known parent for genotyping when pedigree information is limited to help mitigate adverse effects on GPEBV accuracies. Nonetheless, to maximize the precision of genetic assessments, animals with complete pedigree records should be prioritized for genotyping whenever feasible.

### 3.2 Effect of incorporating incomplete pedigree data on genetic parameters

The impact of incorporating incomplete pedigree data on heritability estimates and additive genetic correlations is presented in Table 5. The heritability for MY increased from 0.25±0.03 (SO1) to 0.30±0.03 (SO9), suggesting that missing pedigree data resulted in overestimation of additive genetic variances, thereby inflating heritability estimates. This pattern is consistent with findings indicating that incomplete pedigree information introduces biases in heritability estimates by altering the variance structure, often leading to fluctuations in additive genetic variances (Nilforooshan et al., 2008). In contrast, the heritability for FP declined from 0.19±0.05 (SO1) to 0.06±0.01 (SO9), suggesting that this trait was more susceptible to missing pedigree data than MY, despite a relatively stable additive genetic variance. Similarly, the heritability for AFC exhibited a downward trend, decreasing from 0.18±0.03 (SO1) to 0.10±0.01 (SO9), further highlighting the greater sensitivity of fertility traits to incomplete pedigree data. These findings agreed with studies indicating that fertility traits typically exhibit low heritabilities and are predominantly influenced by environmental factors (Shao et al., 2021; Kgari et al., 2022).

**Table 5:** Heritabilities and correlations for 305 d milk yield (MY), 305 d fat percentage (FP), and age at first calving (AFC) estimated using a three-trait single-step genomic–polygenic model across nine missing pedigree scenarios.

Parameters^b^	Missing pedigree scenario^a^																	
	SO1		SO2		SO3		SO4		SO5		SO6		SO7		SO8		SO9	
	Est.	SE	Est.	SE	Est.	SE	Est.	SE	Est.	SE	Est.	SE	Est.	SE	Est.	SE	Est.	SE
Heritabilities																		
MY	0.25	0.03	0.26	0.04	0.26	0.04	0.26	0.04	0.27	0.04	0.25	0.04	0.26	0.04	0.28	0.04	0.30	0.03
FP	0.19	0.05	0.17	0.05	0.21	0.05	0.18	0.05	0.16	0.06	0.19	0.06	0.12	0.06	0.17	0.06	0.06	0.01
AFC	0.18	0.03	0.15	0.03	0.16	0.03	0.14	0.03	0.10	0.03	0.11	0.03	0.08	0.04	0.11	0.04	0.10	0.01
Genetic correlations																		
MY, FP	- 0.40	0.15	- 0.46	0.17	- 0.37	0.14	- 0.47	0.16	- 0.54	0.20	- 0.44	0.17	- 0.71	0.54	- 0.55	0.33	- 0.91	0.01
MY, AFC	- 0.08	0.12	- 0.19	0.14	- 0.16	0.13	- 0.23	0.15	- 0.16	0.20	- 0.05	0.18	- 0.06	0.33	0.14	0.19	0.09	0.07
FP, AFC	0.26	0.18	0.33	0.22	0.27	0.19	0.35	0.22	0.28	0.34	0.31	0.27	0.22	0.65	0.09	0.36	- 0.44	0.06
Environmental correlations																		
MY, FP	- 0.09	0.04	- 0.08	0.04	- 0.09	0.04	- 0.07	0.04	- 0.06	0.04	- 0.08	0.04	- 0.04	0.05	- 0.05	0.05	- 0.04	0.02
MY, AFC	0.08	0.03	0.10	0.03	0.10	0.03	0.11	0.03	0.09	0.03	0.07	0.03	0.07	0.03	0.03	0.04	0.04	0.02
FP, AFC	- 0.05	0.04	- 0.06	0.04	- 0.05	0.04	- 0.06	0.04	- 0.04	0.04	- 0.05	0.04	- 0.02	0.04	- 0.01	0.04	0.04	0.02
Phenotypic correlations																		
MY, FP	- 0.16	0.02	- 0.16	0.02	- 0.16	0.02	- 0.16	0.02	- 0.16	0.02	- 0.16	0.02	- 0.16	0.02	- 0.16	0.02	- 0.16	0.02
MY, AFC	0.05	0.01	0.04	0.01	0.05	0.01	0.04	0.01	0.05	0.01	0.05	0.01	0.05	0.01	0.05	0.01	0.05	0.01
FP, AFC	0.00	0.02	0.00	0.02	0.00	0.02	0.00	0.02	0.00	0.02	0.00	0.02	0.00	0.02	0.00	0.02	0.00	0.02

^a^ SO1: all available pedigree information; SO2: sire identification removed for all genotyped animals; SO3: dam identification removed for all genotyped animals; SO4: both sire and dam identification removed for all genotyped animals; SO5: no pedigree information for 10 % of animals with phenotypes; SO6: no pedigree information for 20 % of animals with phenotypes; SO7: no pedigree information for 30 % of animals with phenotypes; SO8: no pedigree information for 40 % of animals with phenotypes; SO9: no pedigree information for 50 % of animals with phenotypes. ^b^ Repeated sampling approach of Meyer and Houle (2013).

Additive genetic correlations were also affected by declining pedigree completeness. The genetic correlation between MY and FP became increasingly negative, shifting from -0.40±0.15 (SO1) to -0.91±0.01 (SO9). The additive genetic correlation between MY and AFC fluctuated between -0.08±0.12 (SO1) and 0.09±0.07 (SO9), whereas the FP–AFC correlation showed greater variability, ranging from -0.44±0.06 (SO9) to 0.26±0.18 (SO1). These results indicated increasing instability of estimated genetic correlations as pedigree completeness declined.

In single-step genomic–polygenic evaluation, additive genetic correlations are inferred from covariance information among related individuals represented in the combined pedigree–genomic relationship matrix **(H)**. Progressive removal of pedigree links reduces pedigree-derived covariances contributing to parameter estimation, particularly for non-genotyped animals, while relationships among genotyped animals remain partially preserved through genomic information. Consequently, the observed changes primarily reflect alterations in the available relationship information rather than changes in the underlying biological associations among traits. Similar sensitivity of covariance estimates to incomplete pedigree information has been reported previously (Nilforooshan et al., 2008). Although genomic relationships partially compensated for the loss of pedigree information, genomic data alone were insufficient to fully maintain covariance structure when pedigree connectivity was substantially reduced. These findings indicate that genomic information improves the robustness of genomic–polygenic evaluations but cannot completely replace pedigree information, particularly for traits with lower heritability and weaker genetic connectedness.

### 3.3 Effect of incorporating incomplete pedigree data on genomic–polygenic estimated breeding values

**Figure 1:**
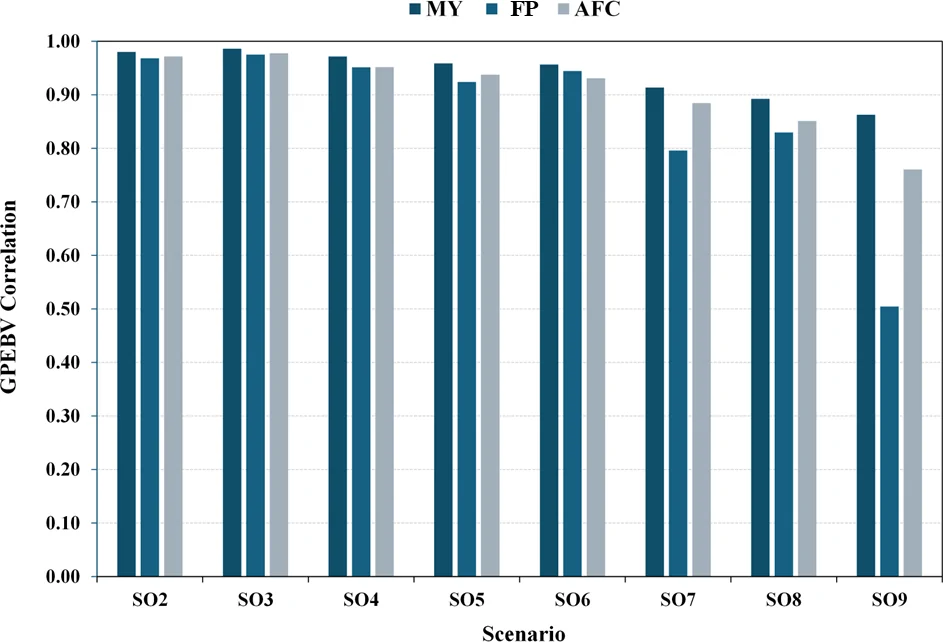
Correlation coefficients between GPEBVs from the complete pedigree scenario (SO1) and GPEBVs from eight scenarios with various patterns of missing pedigree information (SO2 to SO9) for 305 d milk yield (MY), 305 d fat percentage (FP), and age at first calving (AFC). SO1: all available pedigree information; SO2: sire identification removed for all genotyped animals; SO3: dam identification removed for all genotyped animals; SO4: both sire and dam identification removed for all genotyped animals; SO5: no pedigree information for 10 % of animals with phenotypes; SO6: no pedigree information for 20 % of animals with phenotypes; SO7: no pedigree information for 30 % of animals with phenotypes; SO8: no pedigree information for 40 % of animals with phenotypes; SO9: no pedigree information for 50 % of animals with phenotypes.

The impact of missing pedigree on correlations between GPEBVs in the base scenario (SO1) and in alternative scenarios (SO2 to SO9) varied across traits; FP had the most significant decline, followed by AFC, and MY was the least affected (Fig. 1). These differences reflected varying levels of dependence on pedigree-based genetic relationships. Low-heritability traits such as FP and AFC were more sensitive to pedigree completeness, whereas MY, with higher heritability, appeared to be better supported by genomic information. As pedigree information decreased, the accuracy of predicted GPEBVs declined, although the extent of this reduction differed among traits.

When pedigree information was removed only for genotyped animals (SO2 to SO4), GPEBV correlations remained high across traits (0.95 to 0.99), indicating that genomic relationships largely preserved genetic connectedness. In contrast, progressive pedigree loss among animals with phenotypes (SO5 to SO9), which included a greater proportion of non-genotyped individuals, resulted in substantially larger declines in correlations (0.51 to 0.96). This pattern is consistent with the structure of single-step genomic–polygenic evaluation, where relationships among genotyped animals are maintained through genomic information, whereas non-genotyped animals rely primarily on pedigree links. Consequently, severe pedigree incompleteness mainly reduces evaluation stability through loss of relationship information among non-genotyped animals rather than through removal of pedigree information for genotyped individuals alone.

Notably, correlations between GPEBVs from SO1 and SO2 to SO6 remained above 0.90 for all traits (MY: 0.96 to 0.99; FP: 0.93 to 0.98; AFC: 0.93 to 0.98), indicating that genomic data partially compensated for moderate pedigree omissions. However, correlations between GPEBVs from SO1 and SO7 to SO9 declined more substantially, particularly for FP and AFC, highlighting their greater reliance on pedigree-based additive genetic relationships. In particular, correlations between GPEBVs from SO1 and SO9 decreased to 0.86 for MY, 0.51 for FP, and 0.76 for AFC, indicating that genomic information alone was insufficient to fully compensate for missing pedigree data, especially for traits with low heritability (Meyer, 2021).

The pronounced decline in GPEBV correlations in SO7 to SO9 highlights the risks associated with relying solely on genomic selection when pedigree data are highly incomplete because it could compromise selection accuracy and hinder genetic progress, particularly for FP and AFC. As heritability decreases, the capacity of genomic information to compensate for missing additive genetic relationships weakens, leading to greater prediction errors (Masuda et al., 2021; Meyer, 2021). These findings underscore the critical role of pedigree completeness in genomic–polygenic evaluations, as genomic data alone cannot fully replace pedigree-based genetic relationships. Furthermore, the reduced accuracy of GPEBVs in low-heritability traits may compromise the effectiveness of selection programs, ultimately constraining long-term genetic improvement.

Given that data limitations are common in smallholder dairy farming systems, these results indicate that genomic–polygenic evaluations may remain viable when up to 20 % of pedigree data are missing (SO6), as correlations remained above 0.90 for all traits. However, to minimize biases and ensure robust selection decisions, efforts should be directed toward preserving pedigree completeness whenever possible.

### 3.4 Effect of incorporating incomplete pedigree data on animal rankings

The impact of incorporating incomplete pedigree data into genomic–polygenic evaluations became progressively pronounced as the extent of pedigree omissions and selection intensities increased. The most substantial effects were observed under the highest selection intensity (top 5 % of animals), where rank correlations between the base scenario (SO1) and pedigree omission scenarios (SO2 to SO9) markedly decreased from 0.86 to 0.21 for MY, 0.86 to 0.50 for FP, and 0.80 to 0.27 for AFC in sires (Table 6) and from 0.90 to 0.67 for MY, 0.83 to 0.20 for FP, and 0.85 to 0.58 for AFC in cows (Table 7). These findings highlight the critical role of complete pedigree information in maintaining ranking stability and enabling precise selection decisions, thereby notably influencing the accuracy and genetic advancement within animal breeding programs (Nilforooshan et al., 2008; Pimentel et al., 2024).

**Table 6:** Rank correlations between sire GPEBVs from the complete pedigree scenario (SO1) and GPEBVs from eight scenarios with various patterns of missing pedigree information (SO2 to SO9) for 305 d milk yield (MY), 305 d fat percentage (FP), and age at first calving (AFC).

Traits	Sires^b^	Rank correlation coefficients^a^							
		SO1, SO2	SO1, SO3	SO1, SO4	SO1, SO5	SO1, SO6	SO1, SO7	SO1, SO8	SO1, SO9
MY	Top 5 % (57)	0.86 (49)	0.86 (49)	0.79 (48)	0.80 (45)	0.75 (43)	0.63 (36)	0.73 (34)	0.21 (26)
	Top 15 % (172)	0.89 (158)	0.92 (151)	0.85 (146)	0.85 (149)	0.75 (150)	0.57 (136)	0.62 (127)	0.46 (117)
	Top 25 % (286)	0.93 (266)	0.90 (248)	0.86 (247)	0.88 (253)	0.86 (253)	0.73 (233)	0.75 (233)	0.63 (207)
	100 % (1,145)	0.98	0.94	0.93	0.95	0.94	0.87	0.81	0.72
FP	Top 5 % (57)	0.86 (47)	0.82 (44)	0.78 (41)	0.76 (46)	0.73 (41)	0.62 (38)	0.74 (34)	0.5 (17)
	Top 15 % (172)	0.91 (152)	0.83 (144)	0.82 (139)	0.81 (137)	0.82 (133)	0.70 (103)	0.66 (107)	0.17(68)
	Top 25 % (286)	0.91 (256)	0.90 (250)	0.86 (242)	0.81 (243)	0.78 (237)	0.57 (200)	0.63 (197)	0.23 (142)
	100 % (1145)	0.96	0.91	0.89	0.90	0.91	0.75	0.75	0.45
AFC	Top 5 % (57)	0.80 (46)	0.81 (47)	0.72 (43)	0.70 (42)	0.45 (43)	0.52 (38)	0.53 (37)	0.27 (29)
	Top 15 % (172)	0.86 (159)	0.86 (149)	0.79 (144)	0.77 (150)	0.75 (148)	0.64 (136)	0.63 (125)	0.49 (106)
	Top 25 % (286)	0.91 (259)	0.86 (240)	0.78 (235)	0.82 (244)	0.80 (240)	0.69 (218)	0.72 (203)	0.56 (178)
	100 % (1145)	0.97	0.91	0.89	0.91	0.89	0.81	0.75	0.60

^a^ SO1: all available pedigree information; SO2: sire identification removed for all genotyped animals; SO3: dam identification removed for all genotyped animals; SO4: both sire and dam identification removed for all genotyped animals; SO5: no pedigree information for 10 % of animals with phenotypes; SO6: no pedigree information for 20 % of animals with phenotypes; SO7: no pedigree information for 30 % of animals with phenotypes; SO8: no pedigree information for 40 % of animals with phenotypes; SO9: no pedigree information for 50 % of animals with phenotypes. ^b^ Numbers in brackets are numbers of sires.

Sire rankings remained relatively stable when pedigree exclusions were limited to genotyped animals (SO2 to SO4; Table 6). Rank correlations between SO1 and SO2 to SO4 for the top 5 % of sires remained consistently high, ranging from 0.79 to 0.86 for MY, 0.73 to 0.86 for FP, and 0.70 to 0.81 for AFC, indicating that omitting pedigree information exclusively from genotyped animals would have a limited impact. However, ignoring pedigree information from animals with phenotypic records regardless of genotype (SO5 to SO9) resulted in progressive decline in rank correlations. The largest discrepancies were between sire GPEBVs in SO1 and SO9, where correlations were reduced to 0.21 for MY, 0.50 for FP, and 0.27 for AFC. These results illustrate that extensive pedigree omissions notably influence genetic evaluation accuracies, leading to increased variability and uncertainty in animal rankings (Nilforooshan et al., 2008).

A similar trend was observed in cows, with relatively high rank correlations between SO1 and SO2 to SO4, reflecting similar stability to that seen in sires. However, under the most extreme pedigree omission scenario (SO9), cow rank correlations also decreased markedly to 0.67 for MY, 0.20 for FP, and 0.58 for AFC (Table 7). These outcomes confirm that increased pedigree incompleteness negatively affects the accuracy of additive genetic predictions, thereby increasing variability and uncertainty in animal rankings and decreasing the precision of selection decisions in breeding programs.

**Table 7:** Rank correlations between cow GPEBVs from the complete pedigree scenario (SO1) and GPEBVs from eight scenarios with various patterns of missing pedigree information (SO2 to SO9) for 305 d milk yield (MY), 305 d fat percentage (FP), and age at first calving (AFC).

Traits	Cows^b^	Rank correlation coefficients^a^							
		SO1, SO2	SO1, SO3	SO1, SO4	SO1, SO5	SO1, SO6	SO1, SO7	SO1, SO8	SO1, SO9
MY	Top 5 % (893)	0.90 (796)	0.93 (803)	0.86 (770)	0.87 (741)	0.85 (749)	0.74 (673)	0.70 (658)	0.67 (624)
	Top 15 % (2678)	0.93 (2423)	0.94 (2450)	0.90 (2345)	0.87 (2338)	0.87 (2324)	0.77 (2153)	0.73 (2075)	0.69 (1995)
	Top 25 % (2464)	0.93 (4075)	0.94 (4116)	0.90 (3971)	0.89 (3969)	0.88 (3903)	0.79 (3626)	0.75 (3533)	0.71 (3404)
	100 % (17 855)	0.97	0.98	0.96	0.95	0.94	0.89	0.86	0.82
FP	Top 5 % (893)	0.83 (769)	0.93 (806)	0.82 (753)	0.87 (751)	0.71 (691)	0.51 (553)	0.68 (595)	0.20 (300)
	Top 15 % (2678)	0.88 (2312)	0.92 (2337)	0.86 (2209)	0.87 (2271)	0.78 (2142)	0.59 (1797)	0.67 (1897)	0.30 (1216)
	Top 25 % (2464)	0.88 (3914)	0.90 (3924)	0.85 (3740)	0.87 (3836)	0.81 (3670)	0.62 (3173)	0.68 (3242)	0.34 (2319)
	100 % (17 855)	0.96	0.96	0.93	0.90	0.92	0.77	0.79	0.46
AFC	Top 5 % (893)	0.85 (759)	0.88 (766)	0.78 (706)	0.73 (688)	0.70 (660)	0.63 (616)	0.58 (565)	0.58 (491)
	Top 15 % (2678)	0.85 (2328)	0.88 (3550)	0.78 (2205)	0.78 (2190)	0.74 (2173)	0.68 (2016)	0.60 (1852)	0.56 (1622)
	Top 25 % (2464)	0.88 (3981)	0.89 (3993)	0.80 (3781)	0.80 (3748)	0.79 (3755)	0.72 (3485)	0.64 (3242)	0.57 (2911)
	100 % (17 855)	0.96	0.97	0.93	0.91	0.91	0.84	0.79	0.69

^a^ SO1: all available pedigree information; SO2: sire identification removed for all genotyped animals; SO3: dam identification removed for all genotyped animals; SO4: both sire and dam identification removed for all genotyped animals; SO5: no pedigree information for 10 % of animals with phenotypes; SO6: no pedigree information for 20 % of animals with phenotypes; SO7: no pedigree information for 30 % of animals with phenotypes; SO8: no pedigree information for 40 % of animals with phenotypes; SO9: no pedigree information for 50 % of animals with phenotypes. ^b^ Numbers in brackets are numbers of cows.

These findings underscore the necessity of encouraging farmers, particularly smallholder dairy producers in Thailand, to maintain complete pedigree records because comprehensive pedigree documentation is essential for accurate selection decisions. This is particularly critical in the Thai multibreed dairy population, where genetic diversity and crossbreeding complicate pedigree tracking. Maintaining accurate pedigree records is especially important for traits with low heritability because genomic information alone does not fully compensate for missing pedigree relationships. The observed increase in ranking instability with expanding pedigree incompleteness, particularly for FP and AFC, highlights the importance of pedigree completeness to maintain selection accuracy for polygenic traits heavily influenced by environmental factors.

## 4 Conclusions

Incorporating animals with incomplete pedigree data in genomic–polygenic evaluations affected the estimation of additive genetic variances, heritabilities, GPEBV accuracies, and ranking stability, with varying impacts across traits. The additive genetic variance estimates increased for MY and remained relatively stable for FP, suggesting a strong reliance of these traits on genomic information. Conversely, the additive genetic variance estimates for AFC decreased, reinforcing the greater vulnerability of low-heritability traits to missing pedigree data. Heritability estimates increased for MY but decreased for FP and AFC, highlighting the greater dependence of low-heritability traits on pedigree completeness. The deterioration of GPEBV accuracy was accompanied by changes in ranking, particularly for FP and AFC, emphasizing the critical role of pedigree records in maintaining selection accuracy. Among genotyped animals with missing pedigree data, the absence of both sire and dam pedigree records had the highest impact on genomic–polygenic evaluations, leading to greater deviations for estimated genetic parameters and breeding values. Missing pedigree beyond 20 % destabilized genetic evaluations, reducing GPEBV accuracies and consistency of animal rankings, which could lead to reduced genetic progress in the Thai multibreed dairy population. These findings highlight the importance of maintaining complete pedigree records, particularly in smallholder dairy farming systems, where inconsistent record-keeping frequently undermines GPEBV accuracy. Promoting systematic pedigree recording among farmers is crucial for ensuring the reliability of genomic–polygenic evaluations, thereby enhancing selection accuracy and the sustainability of long-term genetic improvement in the Thai multibreed dairy population.

## Data Availability

The data used in this study were obtained from privately owned datasets collected from multiple dairy farms. These data were provided with permission for research use and for the publication of aggregated findings. However, the datasets are subject to restrictions due to data ownership, confidentiality considerations, and existing agreements with the data providers, which prohibit public dissemination. Therefore, the data are not publicly available. Access to the data can be granted by the corresponding author upon reasonable request, subject to prior approval from the respective data owners and compliance with all applicable data-sharing agreements and confidentiality requirements. Only aggregated results are reported in this study to ensure that no individual farm can be identified.
